# Non-linear pyroelectric energy harvesting with commercial multilayer capacitors

**DOI:** 10.1039/d6ta05388b

**Published:** 2026-10-27

**Authors:** Amandine Helt, Omar Ramírez, Asmaa El Moul, Nathalie Valle, Cédric Bamberg, Emmanuel Defay

**Affiliations:** a Luxembourg Institute of Science and Technology (LIST) Esch-sur-Alzette 4362 Luxembourg amandine.helt@list.lu emmanuel.defay@list.lu; b University of Luxembourg Esch-sur-Alzette 4365 Luxembourg

## Abstract

Pyroelectric materials enable the transformation of periodic temperature variations into electrical power, offering a promising and sustainable electricity source. To date, several pyroelectric materials have been tested for waste heat harvesting, including ceramics such as lead scandium tantalate, which showed the highest performance when used in multilayer capacitors. However, these custom-made optimized capacitors are available in limited quantities, which questions their possible use in future large heat harvesters. Here, we investigate the potential of largely available industrially manufactured multilayer capacitors for non-linear pyroelectric heat harvesting. We first present an electrical screening of several of these capacitors, measuring their breakdown voltage, losses and theoretical harvested energy density for a given Olsen cycle. The best sample presents a maximum active energy density of 3.7 J cm^−3^ for a cycle run over a temperature span of 200 °C and under an applied voltage of 450 V. Then, characterization shows that this multilayer capacitor is made of Ca and Zr-doped BaTiO_3_, with a Curie temperature of 10 °C. Finally, the capacitor's performance is evaluated in the context of a prospective heat harvesting device, confirming its potential as a viable candidate for non-linear pyroelectric energy harvesting.

## Abstract 2

A maximum energy density of 3.7 J cm^−3^ has been obtained with a commercially available ceramic multilayer capacitor, which has then been structurally, chemically and electrically characterized.

## Graphical abstract

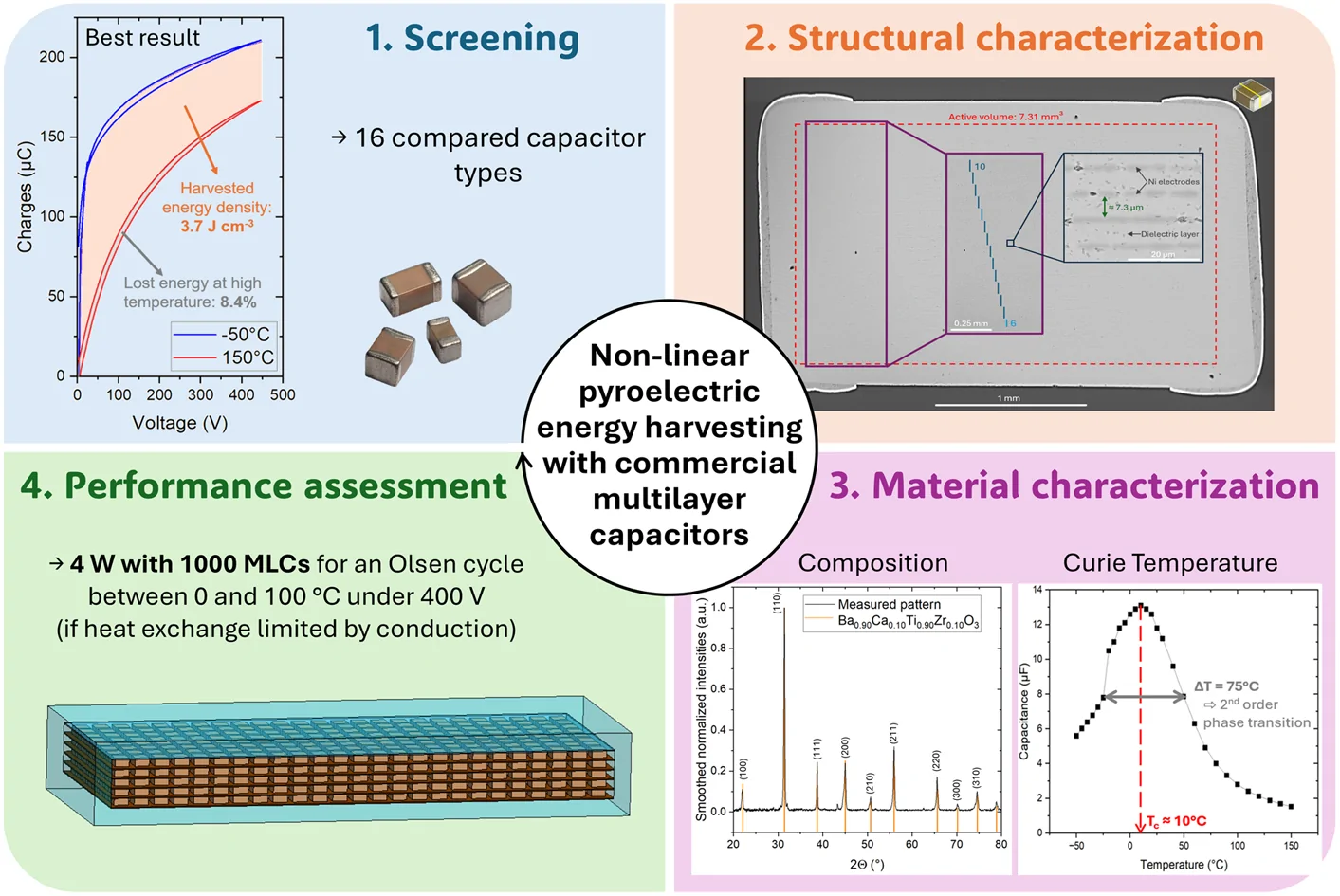

## 1. Introduction

More than 70% of the global primary energy consumption is lost as waste heat, with two-thirds of it being released as low-grade heat (<100 °C), mainly in transportation, power generation systems and industrial processes.^1^ Being able to convert this heat into electricity would help with meeting the growing energy demand while reducing the reliance on non-renewable energy sources such as fossil fuels.^2^

Thermoelectric energy harvesting is for now the predominant commercially available technology for direct heat conversion, with devices transforming spatial temperature gradients into electrical energy using the Seebeck effect.^3,4^ Despite significant improvements, the main limitation of thermoelectric materials remains their low efficiency, especially for low-grade heat harvesting. This can explain the growing interest in pyroelectric energy harvesting,^5^ for which a temporal temperature change is converted into electrical energy using the temperature-dependent intrinsic polarization of some polar dielectric materials.^6^ Indeed, pyroelectric generators present the advantage of being able to work with relatively small temperature variations, even below 100 °C,^7^ while having the potential to reach higher conversion efficiencies than thermoelectric modules.^4,8^

Among all pyroelectric materials, ferroelectrics are of great interest for energy harvesting as they exhibit the largest pyroelectric response, especially if they are used in temperature ranges around their transition temperature (*T*_c_) as polarization decreases rapidly towards zero when approaching this so-called Curie temperature.^6,9,10^ It has been shown that using these materials for non-linear pyroelectric energy conversion, in which the phase transition is driven by both a change in temperature and the application of an electric field, leads to electrical outputs several orders of magnitude larger than with linear energy conversion, meaning in the absence of an applied electric field.^11,12^ Indeed, the strong dependency of polarization on both temperature and electric field makes it possible to perform closed thermodynamic cycles on capacitors made of these materials in order to convert heat into electricity.^13^

A recent study has presented lead scandium tantalate (PST) as one of the most promising materials for non-linear pyroelectric energy conversion, showing the highest reported energy density of 4.4 J cm^−3^ per cycle with a single capacitor,^14^ as well as a Curie temperature at zero applied field close to room temperature, making it even more efficient for low-grade heat harvesting.^15^ Lead zirconate titanate (PZT) based materials also gained attention in the past years due to their tuneable ferroelectric properties and transition temperatures, which can be optimized by doping for example with La^3+^ ^16–18^ or Sn^4+^. ^19,20^ However, these materials are based on lead, which is a toxic element for both human health and the environment.^21^ In addition, PST is made of relatively expensive and scarce elements,^22^ making its use for upscaling quite challenging. That is why other studies tend to focus on lead-free ceramics made of more affordable and abundant elements such as barium titanate (BTO), whose properties can be enhanced using dopants like Zr^4+^, Sr^2+^ or Ca^2+^.^23–26^

Most of these materials have shown enhanced heat conversion performance when being used in the form of thin films.^27–29^ However, despite allowing the application of large electric fields and enabling fast heat transfer thanks to their small thickness,^30,31^ thin films show low amounts of stored energy because of their low thermal mass.^30^ Furthermore, standard methods used to measure temperature changes or produced electrical currents and thus assess their performance are complex due to their small sizes and the general presence of substrates.^28,32^ On the other hand, bulk materials are attractive thanks to their large volume, leading to higher amounts of stored charges. Nevertheless, their large thickness leads to restricted heat exchanges, and reduces both the magnitude of electric field (for the same applied voltage) and breakdown field, as thicker dielectrics are more likely to present internal defects that can cause electrical failure.^33^ One way to combine the advantages of both films and bulk materials is to use multilayer capacitors (MLCs), composed of thin dielectric layers (typically from a few to several tens of microns in thickness) interdigitated with electrodes that are alternately connected on opposing sides by conductive terminals. The metallic electrodes also present the advantage of conducting heat from inside to outer surfaces, improving thermal exchange with respect to bulk materials.^5,34^

MLCs optimized for low-grade heat harvesting, meaning based on ferroelectric materials presenting *T*_c_ around room temperature and with a geometry that enhances heat exchanges, are made on demand in limited quantities and are thus relatively expensive. This limits the future possibility to use these components in large heat harvesting devices. However, ceramic multilayer capacitors are also manufactured industrially to be used in electronic devices, and are thus commercially available in large quantities. These components are usually made from BTO-based ceramics and come in different standardized sizes,^35^ ranging from less than 0.5 mm to a few millimetres in length. Some studies have shown the possibility to create a temperature gradient based on the electrocaloric effect using this type of capacitor.^36,37^ As the electrocaloric effect is the reverse effect of the pyroelectric one, good electrocaloric materials are also good pyroelectric materials.^11,38^

In this work, we performed a comparative screening of several commercial multilayer capacitor variants, evaluating their losses and electric energy density harvested from heat to assess their potential for non-linear pyroelectric heat harvesting. A maximum active energy density of 1.3 J cm^−3^ deduced from polarization measurements of an Olsen cycle performed between 25 °C and 180 °C under 250 V was obtained with one of the capacitors. The latter has been further characterized, investigating its internal structure with SEM imaging, its composition through EDX, SIMS and XRD measurements, and its Curie temperature using the temperature dependence of the capacitance. We show that the dielectric material is (Ba,Ca)(Ti,Zr)O_3_, with a Curie temperature of 10 °C. By optimizing the pyroelectric cycle around the phase transition and pushing the voltage up to 90% of the breakdown limit, we show an increase of the energy density up to 3.7 J cm^−3^, for a temperature span of 200 °C. Finally, direct Olsen cycles were implemented to confirm the order of magnitude of the energy densities obtained from polarization measurements. 1.1 J cm^−3^ were measured with both direct and indirect techniques, for an Olsen cycle performed at 400 V between 0 and 100 °C. We also assessed the capacitor's performance based on its heat exchange properties in the context of a future heat harvesting device. The calculations show a theoretical electrical power of 4 W per cycle obtained with a thousand capacitors, which is on the same order of magnitude as a recent study which uses PST MLCs.^7^ Our work thus demonstrates the potential of commercial lead-free multilayer capacitors for non-linear pyroelectric energy harvesting, owing to their large commercial availability.

## 2. Materials and methods

### 2.1. Capacitors screening

The different studied MLCs are commercially available components. The manufacturer references can be found in SI Table 1. In this study, several parameters are compared, as represented in the capacitor's name whose form is: capacitor number – dielectric type – size – capacitance – voltage. The dielectric type reflects the permitted capacitance change within the recommended operational temperature range.^39^ The size is defined by the length and width of the capacitor in hundredths of an inch. In this study, capacitors' dimensions have been chosen to balance handling and characterization convenience with sufficient compactness, aiming at efficient thermal transfer in future heat exchanger devices. Then, the capacitance is usually measured as the amount of charge that can be stored in the capacitor for no or very low voltage, at room temperature.^40^ Finally, the recommended voltage corresponds to the maximum continuous voltage the capacitor can withstand safely, while avoiding poling or hysteresis.^36^ These two effects could compromise the capacitor's function in a standard electronic application, but not in the case of pyroelectric energy harvesting. It is thus possible to apply higher voltage, as long as it remains below the breakdown threshold.

### 2.2. Electrical characterization method for capacitors testing

The electrical characterization of all samples has been performed using an aixACCT TF Analyser 2000E (aixACCT Systems, Germany). All measurements were performed at 1 Hz with a triangular waveform. The total dielectric layer area input in the software is fixed at 1 cm^2^ for all samples, meaning that the polarization given by the device in µC cm^−2^ is equivalent to a geometry-independent quantity of charges in µC, which enables a more accurate comparison of the samples whose structure is unknown. Unipolar charges–voltage (*QV*) loops were measured at both 25 °C and 180 °C, with applied voltages from 25 V up to the breakdown limit (with 25 V steps). A bipolar loop was done in between two unipolar loops to erase the remnant polarization. The high temperature has been defined to be higher than BTO's Curie temperature, which is known to be around 120 °C,^41^ as the harvested energy is larger when the pyroelectric cycle lies in the phase transition region.^14^ In addition, using a high temperature for the screening step enables finding a capacitor that will only show small leakage when being cycled up to lower temperature.

### 2.3. Structural, chemical and electrical characterizations of the best capacitor variant

The internal structure of the capacitor was investigated using a Quanta 200 Field Emission Gun Scanning Electron Microscope (FEG SEM) from Philips-FEI. Then, a Genesis XM 4i Energy Dispersive X-ray (EDX) spectrometer was used to determine the elemental composition of the electrodes, terminals and dielectric material. For the latter, Secondary-Ion Mass Spectrometry (SIMS) using a CAMECA SC-Ultra instrument, and X-ray Diffraction (XRD) using a Brucker D8 diffractometer in the 20–80° 2*θ* range, were also used to fine-tune the composition. XRD peaks were indexed using the PDF file number 00-063-0610.

In order to know the Curie temperature of the dielectric material, the evolution of the capacitance at zero electric field depending on the temperature was measured using a multimeter in capacitance meter mode (FLUKE 175 True-RMS digital), while the sample was heated using a Linkam temperature control stage (THMS600, Linkam Scientific Instruments, United Kingdom) with active cooling by liquid nitrogen (LNP95).

The energy increment graph of Fig. 4b is based on bipolar *QV* loops measured every 10 °C between −50 and 140 °C with the aixACCT system, at a frequency of 0.1 Hz and under a current of 10 mA. The positive part of these loops used for the energy density calculation is available in SI Note 5, Fig. S5-1. Other energy density values mentioned in the first part of Section 3.3 were calculated from bipolar *QV* loops measured at 1 Hz.

Direct Olsen cycles were performed under different voltages ranging from 50 to 450 V (with 50 V steps).^13,42^ The sample was first charged isothermally at 0 °C, then heated up to 100 °C under constant voltage, before being fully discharged at constant high temperature and cooled down back to 0 °C. The Linkam stage mentioned previously has been used for temperature control, associated with a Keithley 2410 source measure unit (Tektronix, United States) for voltage generation, both controlled by a Python script developed for automatic operation and data acquisition.

## 3. Results and discussion

### 3.1. Capacitors testing

The primary goal of this section is to investigate whether or not some commercially available capacitors could be good candidates for non-linear pyroelectric energy harvesting. For this application, larger permitted capacitance changes should enable reaching larger amounts of harvested energy. That is why most of the selected capacitors are from the Y5V dielectric type,^39^ as identified for electrocaloric capacitors,^36^ while some others have been chosen for comparison. Another goal of this section is to determine if the different parameters have any influence on the capacitor's pyroelectric performance. In total, sixteen capacitor variants have thus been selected, with different dielectric types, sizes, capacitance values and maximum recommended voltages (see explanation in Section 2.1).

All capacitor types have been compared based on three parameters that were measured following the electrical characterization method detailed in Section 2.2. First, the breakdown voltage was measured at 180 °C. As the internal structure of the capacitors, and especially the thickness of the dielectric layers, is not known, the applied electric field is also unknown. To compare the samples' behaviour under an equivalent applied electric field, we thus used a certain percentage of their respective breakdown voltages. Then, unipolar charges–voltage (*QV*) loops were taken at an applied voltage of around 25% of the breakdown threshold (*V*_25%_) and a temperature of 180 °C, in order to calculate the losses (comprising dielectric losses and leakage current) at high temperature. The latter are defined as the ratio between the area of the unipolar loop, representing the lost energy, and the area above the charge curve (lower curve), corresponding to the energy stored in the capacitor when fully charged. An example corresponding to C16 – Y5V – 1210 – 10 µF – 16 V is given in Fig. 1a. Finally, the energy density (*N*_d_, in J cm^−3^) that could be obtained from an Olsen cycle performed between 25 °C and 180 °C, from 0 V to *V*_25%_, has been worked out from the unipolar *QV* loops taken at these temperatures, as shown in Fig. 1b. Indeed, an Olsen cycle consists of two isofield legs (here with zero applied voltage in leg DA and 125 V in leg BC) and two isothermal legs (here 25 °C in leg AB and 180 °C in leg CD).^13^ The harvested energy thus corresponds to the area between the bottom curve (charge curve) of the low-temperature loop and the upper curve (discharge curve) of the high-temperature one. The energy density was then calculated as the ratio of the energy value to the total volume of the capacitor. The *QV* loops for all screened capacitors can be found in SI Note 1, Fig. S1-1.

**Fig. 1:**
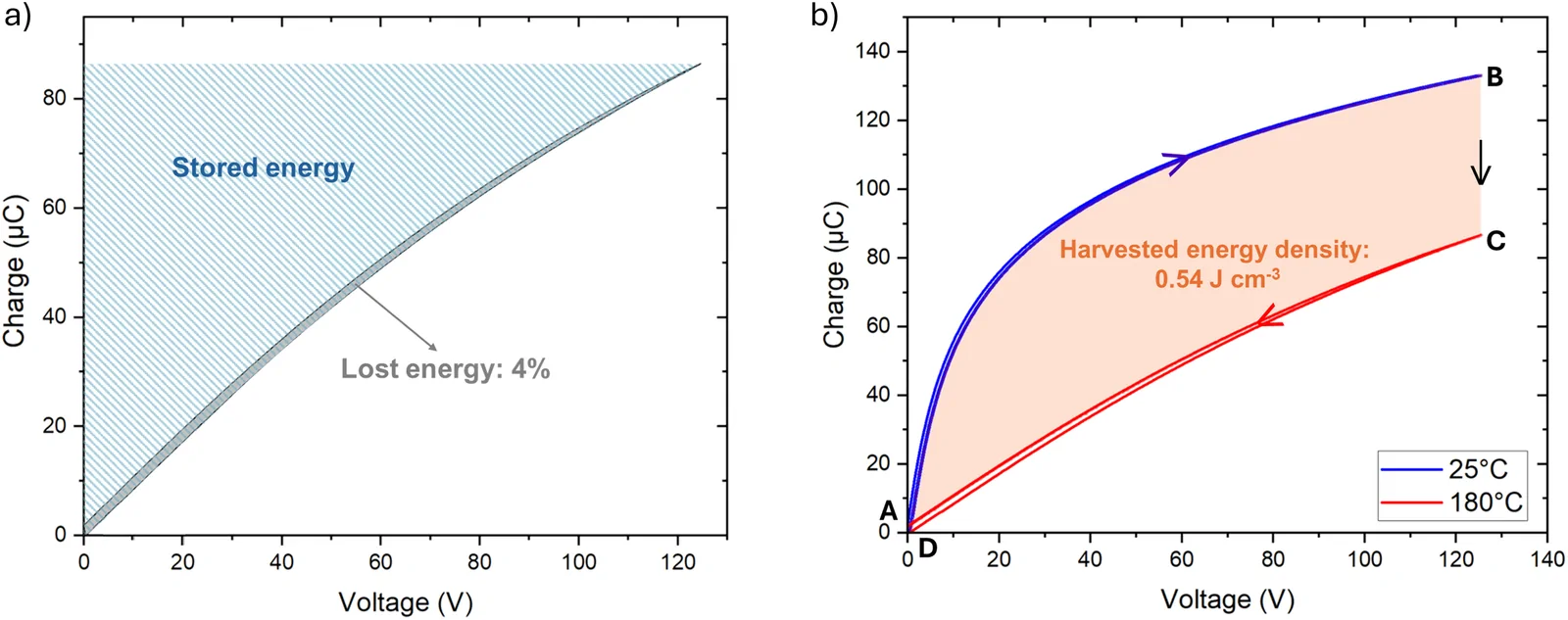
Electrical characterization results obtained for capacitor C16 – Y5V – 1210 – 10 µF – 16 V with aixACCT system, for a frequency of 1 Hz. (a) Unipolar *QV* loop measured at 180 °C for an applied voltage of 125 V. The blue hatched zone represents the energy stored in the capacitor when fully charged, and the inside area of the loop, in grey, corresponds to the lost energy. (b) Unipolar *QV* loops measured from 0 to 125 V at 25 and 180 °C, in blue and red respectively. The letters ABCD refer to the different steps of the corresponding Olsen cycle: A–B: MLC charged up to 125 V at 25 °C, B–C: MLC kept at 125 V while the temperature increases up to 180 °C, C–D: MLC discharged at 180 °C, D–A: MLC cooled down to 25 °C at zero voltage. The coloured area corresponds to the energy density harvested in one cycle.

The values of these three parameters for all studied capacitors are displayed in Fig. 2a, where samples are sorted by ascending value of breakdown voltage, and can be found in SI Table 1. It can be seen that samples showing the smallest losses are predominantly Y5V dielectrics. Two exceptions emerge: sample C5 – Y5V – 1206 – 22 µF – 6.3 V exhibits lower energy density as well as larger losses than two X7T samples (C6 and C7), and sample C8 – Y5V – 1210 – 22 µF – 10 V is among the samples showing the largest losses with no calculable energy density. This shows that Y5V capacitors are not all similar, and that this screening step was essential.

**Fig. 2:**
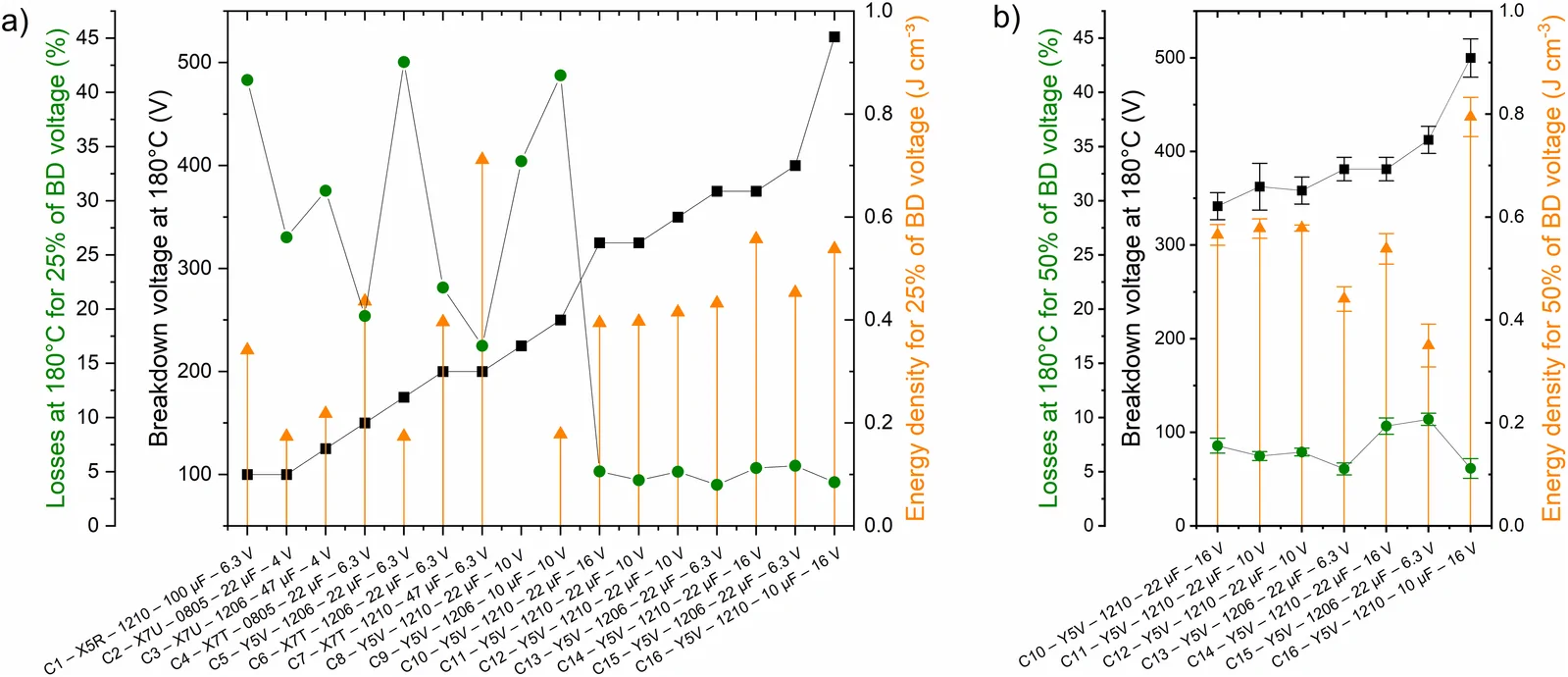
Electrical characterization results obtained for the studied samples, measured at a frequency of 1 Hz with aixACCT system. Black squares correspond to the breakdown voltage at 180 °C. Green circles represent the losses at 180 °C for an applied voltage of around 25% or 50% of the breakdown voltage, for (a) and (b) respectively. Orange arrows show the theoretical energy density obtained for an Olsen cycle performed between 25 °C and 180 °C, from 0 V to 25% or 50% of the breakdown voltage, again for (a) and (b) respectively. In (b), the symbols represent the average values measured over four different samples, while the error bars correspond to the standard deviation of this population. The C16 – Y5V – 1210 – 10 µF – 16 V samples were taken from two different batches in order to assess batch-to-batch variations.

Among all studied capacitors, variants 10 to 16 show the largest energy density values, ranging from 0.4 to 0.6 J cm^−3^, while presenting low losses of around 5%. To identify the best candidate for a deeper study, these capacitor types were re-evaluated under a higher electric field to determine which of them would maintain good properties while being pushed further, meaning the highest energy density accompanied by low losses. A statistical study was conducted in order to assess sample-to-sample variations. For each capacitor type, *QV* loops were measured on 4 samples, first at 25 °C from 25 V up to 50% of the previously determined breakdown voltage, and then at 180 °C from 25 V until breakdown (see examples for one sample of each type in SI Note 1, Fig. S1-2). The losses at high temperature as well as the energy density harvested from an Olsen cycle between 25 and 180 °C were calculated for each sample at the lowest measured breakdown voltage, as explained at the beginning of this section. Then, the average values *µ* of each parameter (breakdown voltage, losses and energy density) and the so-called sample standard deviation given by 
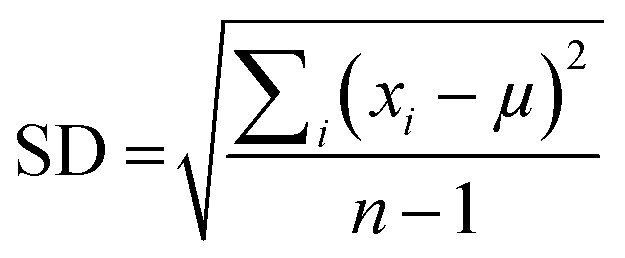
 were calculated for each *n*-samples population. They can be found in SI Table 1 and are represented in Fig. 2b, by the symbols and the error bars respectively. One can conclude that all variants present repeatable behaviours, with error ranges for the breakdown voltage between 3% and 7%, for the losses at high temperature between 5% and 17%, and in the energy density from 1% to 12% of the average values.

In addition to showing losses of only 5.3 ± 0.9%, which is the second lowest value among the sixteen studied capacitor types, C16 – Y5V – 1210 – 10 µF – 16 V also presents the highest energy density value measured for 50% of its breakdown voltage, namely 0.79 ± 0.04 J cm^−3^. This performance makes it the best candidate of this selection for non-linear pyroelectric energy harvesting.

### 3.2. Structural and chemical characterization

A deeper characterization has been performed on C16 – Y5V – 1210 – 10 µF – 16 V. Details of the different measurements can be found in Section 2.3.

The structure of the multilayer capacitor has been investigated using SEM (Fig. 3a), in order to determine the number (*n*), surface area (*S*) and thickness (*t*) of the dielectric layers. The active volume (*V*_a_) of the capacitor, defined as the volume in which the interdigitated electrodes overlap and that actually participates in energy storage, was then calculated.^43^ The first two parameters are required when calculating the polarization (*P*) within the MLC from the charge value (*Q*) given by the aixACCT system, as 
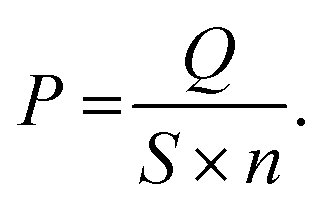
 The thickness of a layer is necessary when calculating the electric field (*E*) across a dielectric layer using the applied voltage (*V*), as 
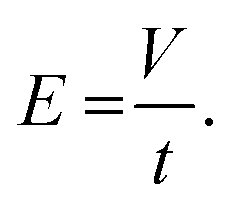
 The active volume is meanwhile needed to calculate the real harvested energy density (*N*_d_), as introduced in the previous section.

**Fig. 3:**
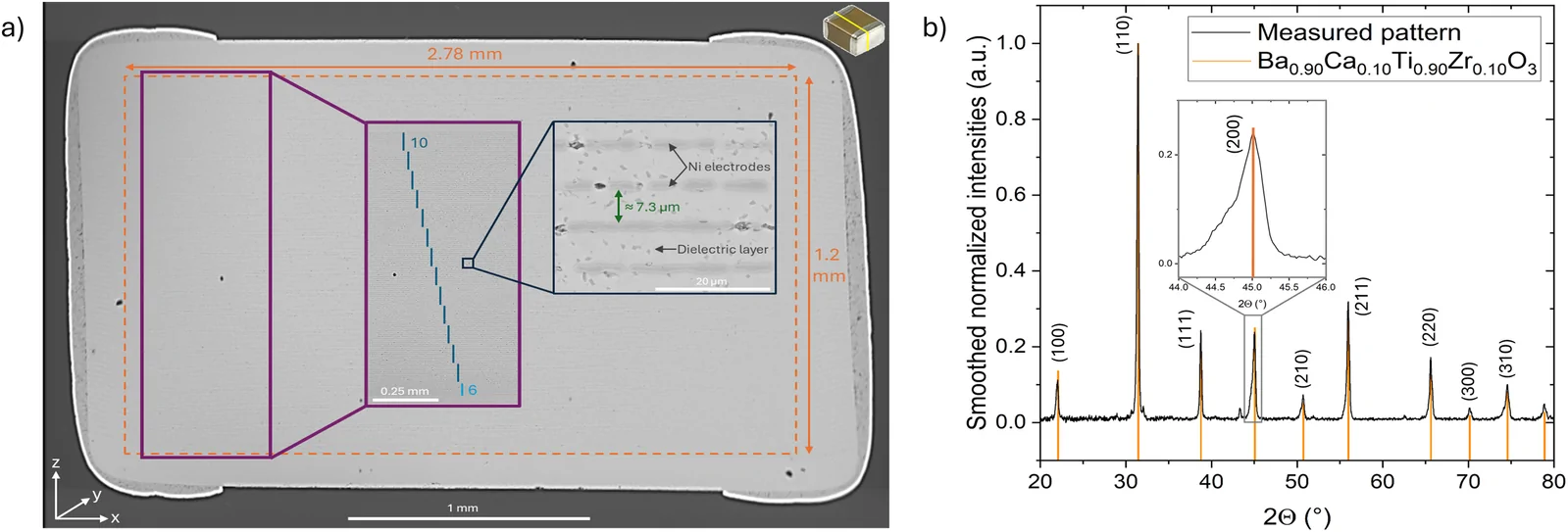
Structural and chemical characterization results of capacitor type C16 – Y5V – 1210 – 10 µF – 16 V. (a) Backscattered electron (BSE)-SEM image of the capacitor cut along the (*x*–*z*) plane, as represented in yellow in the top right corner. The purple-framed zoom-in shows the number of dielectric layers while the blue-framed enlargement gives a better view of their thickness. (b) In black, XRD pattern taken on a dielectric layer of the capacitor. The orange peaks correspond to the best-fitting composition [PDF file 00-063-0610].

As shown in Fig. 3a, C16 – Y5V – 1210 – 10 µF – 16 V is composed of 136 dielectric layers, located along plane (*x*–*y*), with an average thickness of 7.3 µm per layer. The active area of each layer is 6.09 mm^2^, with a length of 2.78 mm (Fig. 3a, axis *x*) and a width of 2.19 mm (see SI Fig. 2, axis *y*). Considering a total height along axis *z* of 1.2 mm as shown in Fig. 3a, it is possible to calculate an active volume of 7.31 mm^3^. In the case of this capacitor, the active volume accounts for 56.2% of the total volume, calculated to be 13 mm^3^ (3.3 × 2.58 × 1.52 mm). Knowing these parameters, the energy density of the active part of sample C16 – Y5V – 1210 – 10 µF – 16 V, as well as the applied electric field can now be calculated. At 50% of the breakdown voltage, corresponding to 340 kV cm^−1^, *N*_d_ reaches 1.3 ± 0.1 J cm^−3^ for a temperature difference of 155 °C. A deeper discussion on the energy density will be provided in the next section.

Besides, the composition of the capacitor has been investigated to get some insights into the materials in use. To do so, EDX measurements were performed on the same surfaces as those studied with SEM. As it can be seen in SI Note 3, electrodes are composed of nickel, while terminals are mainly made of copper and covered with a layer containing nickel and tin. The goal of this layer deposited by electroplating is to improve the terminal's electrical conductivity and facilitate soldering.^44^ However, it was not possible to precisely determine the composition of the dielectric material due to very close characteristic energy values of the different expected elements. In fact, the K-α and L-α emission lines of titanium (Ti) have almost the same energy values as the L-α line of barium (Ba) and the K-α line of oxygen (O), respectively,^45^ which can explain why only these two elements are detected.

A SIMS analysis was thus performed to determine the elements which constitute the dielectric material. As extracted from SI Note 4, the expected Ti, Ba and O were found in the material, confirming that this capacitor is BTO-based. Both zirconium (Zr) and calcium (Ca) were detected, which suggests that this material is (Ba,Ca)(Ti,Zr)O_3_ (BCTZ).

As the SIMS technique does not give any indication of the proportion of each element in the material, XRD was performed at room temperature and, by implementing the known elements in the Diffrac.EVA software, a good match was obtained for composition Ba_0.90_Ca_0.10_Ti_0.90_Zr_0.10_O_3_, as shown in Fig. 3b [PDF file 00-063-0610]. However, it has to be noted that the real composition could differ from the identified one, as XRD diffractograms for all BCTZ compositions (and more largely for BTO-based materials) are very similar, which makes them difficult to differentiate. For example, Panda *et al.*^24^ presented XRD patterns for the composition Ba_0.85_Ca_0.15_(Ti_1−*x*_Zr_*x*_)O_3_, with *x* from 0.05 to 0.30, which are almost identical to our measured pattern in terms of peak intensity and location. When looking more closely between 44° and 46°, a peak at 45° can be seen in Fig. 3b, which would correspond to a Zr content of 0.20 according to Panda *et al.* However, the Ca content in their study is higher than that obtained from our XRD measurement (0.15 and 0.10, respectively), which could also explain the shift of the peak location for *x* = 0.10 towards higher angles, as suggested in ref. 23 and 46.

From this analysis, we can nevertheless conclude that the dielectric material in these MLCs is (Ba,Ca)(Ti,Zr)O_3_ with a majority of Ba compared to Ca (Ba/Ca ∼ 9/1) and a majority of Ti compared to Zr (with Ti/Zr between 9/1 and 8/2). This could partially explain the superior pyroelectric properties exhibited by C16 – Y5V – 1210 – 10 µF – 16 V compared to the other variants, as Ca and Zr are known to improve BTO's performance. Indeed, Zr^4+^ is commonly used to replace Ti^4+^ on B sites as it broadens the transition peak,^23^ which is targeted in commercial devices used *e.g.* in electronics, in which a strong phase transition is not desirable as it would change the capacitor's properties. It has also been shown to be chemically more stable than titanium,^25^ and significantly decreases BTO's transition temperature from 120 °C to below room temperature depending on the proportions,^23^ which is required for low-grade heat harvesting. Ca^2+^ meanwhile takes the position of Ba^2+^ atoms on A sites, and improves the material's resistance by trapping oxygen vacancies, which is one of the most important degradation mechanisms in BTO.^47^ Several studies have found Ba/Ca ∼ 9/1 to be the optimum ratio,^23,25,46^ as too much of it would in fact create migration paths for the vacancies and promote breakdown. A moderate Ca concentration also stabilizes the phase transition over a wider temperature range and lowers dielectric losses.^25,46^

### 3.3. Electrical characterization

The composition of C16 – Y5V – 1210 – 10 µF – 16 V suggests a drastic change in transition temperature compared to that of pure BTO, especially due to the Zr content, as it has been shown by Panda *et al.* with *T*_c_ values going from 98 °C for *x* = 0.05 to below room temperature for *x* ≥ 0.20.^24^ The permittivity of the material showing a maximum at *T*_c_ and being directly linked to the capacitance value,^48^ the evolution of this last parameter at zero electric field depending on the temperature has been measured and is displayed in Fig. 4a. A peak is visible at 10 °C, corresponding to the Curie temperature. According to Panda *et al.*, it should be aligned with a Zr content around 0.20. However, as mentioned previously, the different Ca contents between their study and our XRD match could also explain the shift in *T*_c_ for the same Zr content. This nevertheless confirms our previous conclusion of Ti/Zr being between 9/1 and 8/2, and suggests that Ba/Ca could be between 9/1 and 8.5/1.5. It can also be noted that the transition region is relatively broad, which is typical of second-order phase transitions as can be expected in BCTZ due to the addition of Zr.^23^

**Fig. 4:**
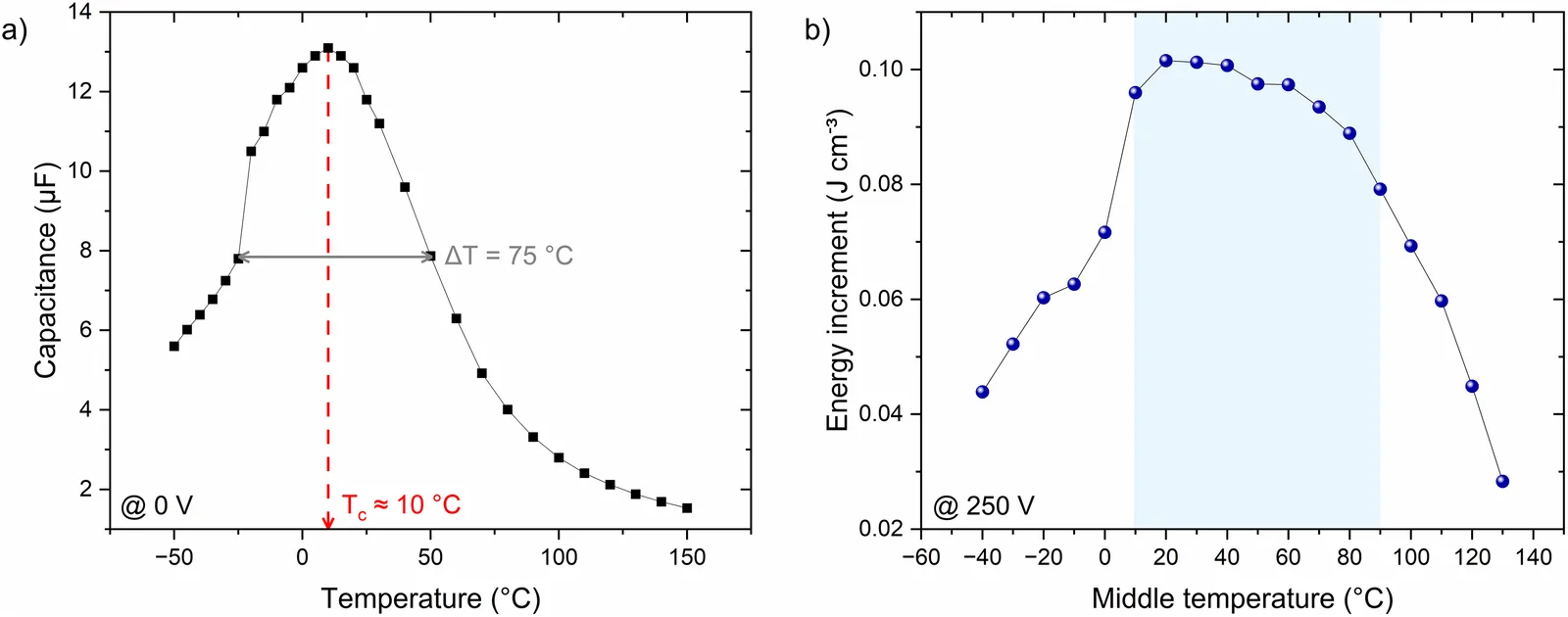
(a) Capacitance *vs.* temperature measurement of capacitor C16 – Y5V – 1210 – 10 µF – 16 V under zero electric field. The peak's maximum gives the Curie temperature value. (b) Temperature-dependence of the energy increment for 20 °C temperature spans, from – 50 to 140 °C, under 250 V (corresponding to 50% of the breakdown limit). Each point corresponds to the middle temperature of the span. The blue zone highlights the temperature range where the highest increments can be found. Values have been extracted indirectly from the *QV* loops presented in SI Note 5.

Several studies have suggested that taking advantage of the ferro- to para-electric phase transition of the material should enable increasing the amount of harvested energy,^14,42,49^ probably due to the increase in pyroelectric coefficient around this temperature.^49^ To assess the impact of the transition on our commercial multilayer capacitors, we calculated the energy density for 20 °C temperature spans based on *QV* loops measured from −50 to 140 °C at 50% of the breakdown limit, meaning under 250 V. The loops can be found in SI Note 5, while the temperature-dependent curve of the energy increments is displayed in Fig. 4b. From the latter, one can observe the largest increments for points between 10 and 90 °C, as represented by the blue zone, meaning over a temperature range between 0 and 100 °C, as each point corresponds to the middle temperature of the 20 °C temperature span. This confirms that running a cycle over the transition region should enable using the full energy potential of the capacitor, and even suggests that the optimum cycling temperature (here around 40 °C) is higher than the Curie temperature. Hence, we performed a cycle between −20 and 130 °C, lying around the optimum region in blue, keeping a voltage of 250 V and a temperature span of 150 °C, as for previous cycles. The harvested energy density value rose from 1.3 to 1.8 J cm^−3^ (a 40% increase), which confirms the benefit obtained from shifting the cycle towards the transition area. By considering the full temperature range of Fig. 4b, we measured another increase of around 25%, as the active energy density reaches 2.1 J cm^−3^.

To assess the full potential of the C16 capacitor variant, we pushed one sample to 90% of its breakdown limit, applying 450 V (616 kV cm^−1^), with a temperature span of 200 °C centered around the optimum cycling temperature (−50 to 150 °C). Under these conditions, we obtained a maximum active energy density of 3.7 J cm^−3^ (see cycle in SI Note 5, Fig. S5-2). The best result in the literature for MLCs was obtained so far with PST, showing a maximum active energy density of 4.4 J cm^−3^ at 195 kV cm^−1^ for a temperature span of 175 °C,^14^ while lead-free BST reaches 3.7 J cm^−3^ under 300 kV cm^−1^ and for a Δ*T* of 170 °C.^42^ Therefore, our results show that commercially available MLCs that are initially not meant to be used for waste heat harvesting can be nearly as good as bespoke capacitors regarding energy density, and that they are very well suited to scale-up.

Finally, direct Olsen cycles have been performed on our Linkam stage (see Section 2.3) over the optimum temperature span suggested in Fig. 4b, meaning between 0 and 100 °C, which presents the advantage of being easy to maintain and could be used with any heat-carrying fluid, notably water. Energy values have been measured as described in Section 2.3, and the resulting energy densities calculated using the active volume *V*_a_ are displayed in Fig. 5, showing values from 0.7 J cm^−3^ at 50 V to 1.2 J cm^−3^ at 450 V. An indirect measurement using *QV* loops was also performed at 400 V using the same temperature span and frequency as those used for the direct measurements (see SI Note 6). The obtained energy densities are 1.11 and 1.13 J cm^−3^ for direct and indirect cycles, respectively, which confirms the approach.

**Fig. 5:**
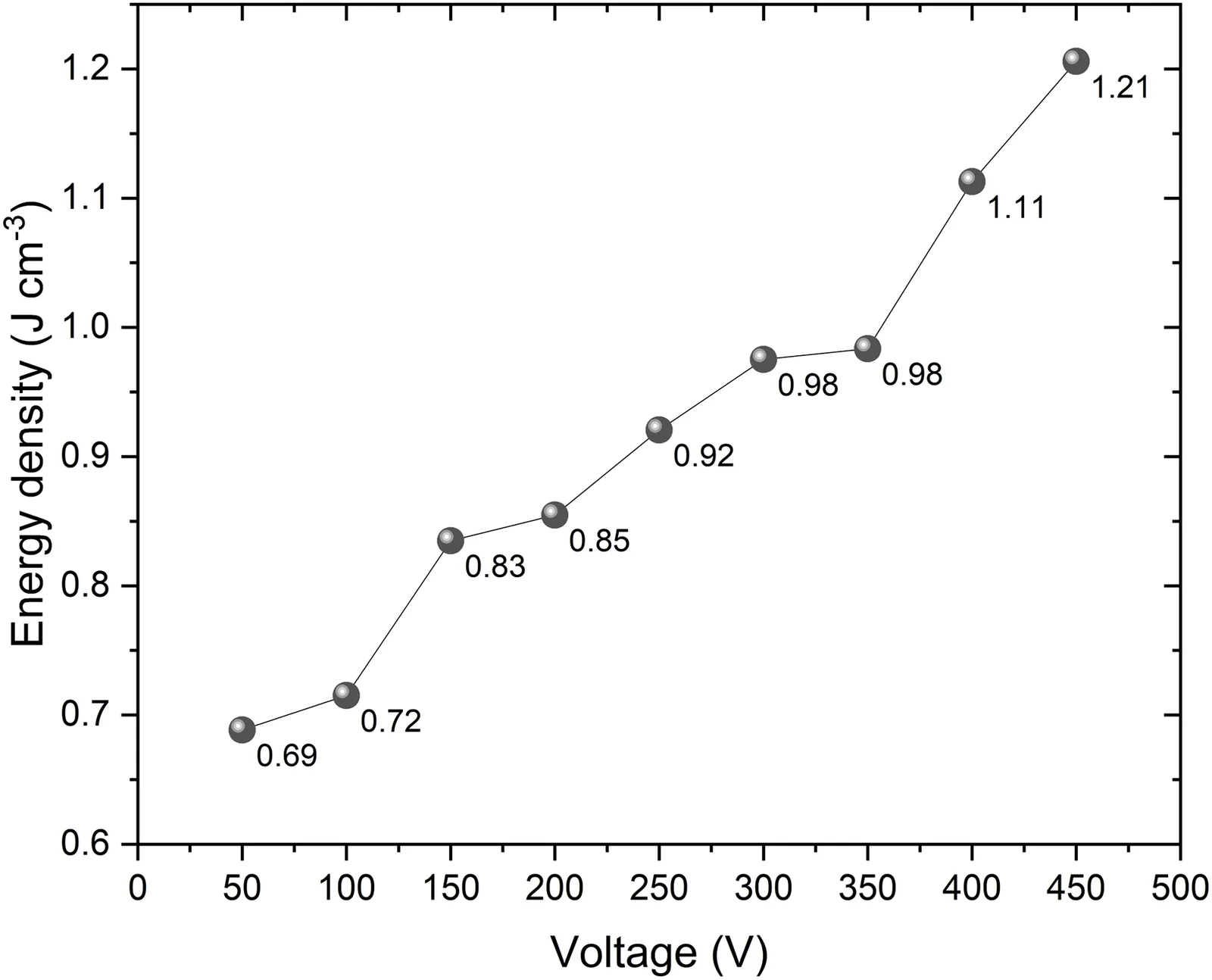
Energy density values obtained through direct Olsen cycles performed between 0 °C and 100 °C on sample C16 – Y5V – 1210 – 10 µF – 16 V, under different applied voltages.

### 3.4. Discussion on future performances of a non-linear pyroelectric generator

This last section aims to assess the performance of C16 – Y5V – 1210 – 10 µF – 16 V MLC for future use in a heat harvesting device. Large heat exchange between the MLC and the heat-carrying medium is crucial in pyroelectric energy harvesting. Indeed, reaching the highest temperature difference (Δ*T*) in the smallest amount of time is necessary to increase the electrical power output given by *P*_e_ = *N* × *f*, with *f* the cycle frequency and *N* the energy. It is thus important to know how long it would take for the pyroelectric material to heat up or cool down depending on its thermal properties, in order to adapt the cycle duration and find a balance between fully exploiting the capacitor's potential and maintaining a high power value. The intrinsic thermal time scale (*τ*_int_) characterizes the thermal relaxation time of a body for conduction. In the simplified case of the parallel-plate capacitor geometry, it is given by 
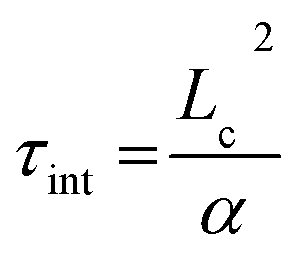
 with *α* the thermal diffusivity and *L*_c_ the characteristic length given by the smaller thickness of the dielectric material.^50,51^ In multilayer capacitors, the latter can be approximated by the sum of the thicknesses of all the layers. In the case of C16 – Y5V – 1210 – 10 µF – 16 V, which contains 136 layers of around 7.3 µm each, the total dielectric thickness is close to 1 mm. According to the literature,^52,53^ the thermal diffusivity of doped BTO ceramics is close to 1 × 10^−6^ m^2^ s^−1^, meaning that *τ*_int_ ≈ 1 s. We saw in the previous section that the cycle measured between 0 and 100 °C for an applied voltage of 400 V gives an energy density of 1.11 J cm^−3^, corresponding to an energy value of 8.13 mJ. Considering a thermal relaxation time of 1 s for both heating and cooling steps, the calculated power output would be 4.1 mW per capacitor. This means that a device composed of 1000 MLCs could generate around 4 W, in the ideal case of heat exchange being limited by conduction only. In comparison, Song *et al.*^7^ recently demonstrated the generation of 1 W of electric power with a generator composed of 255 PST MLCs (corresponding to around 10 cm^3^ of material) running at a frequency of 0.2 Hz. The performance of this generator, like most of the current devices, is still limited by convection even though conduction starts being restrictive too, as suggested by a Biot number of 0.16, the calculation of which is detailed in SI Note 7. Under the same operating conditions, we can calculate that 1000 of our studied MLCs, for a material volume of around 13 cm^3^, should generate 1.6 W. This is a more realistic value taking into account that current prototypes still operate in convective regimes, but which highlights again the competitiveness of commercially available multilayer capacitors for scaling-up pyroelectric harvesting systems.

To further improve the performance of such harvesting devices, it is necessary to reach a conduction-limited regime, by first increasing convection. This could be done by increasing the flow rate, decreasing the gap between capacitors, and using a less viscous fluid with a larger thermal conductivity, as suggested by the Biot number and convective heat-transfer coefficient formulas given in SI Note 7. Once the heat exchange becomes limited by conduction (meaning at large convection), optimizing the capacitor's geometry and material would become the critical factor. Indeed, as suggested by the thermal time scale equation mentioned previously, a thinner capacitor composed of a dielectric material with a larger thermal diffusivity would exchange heat faster, and thus lead to a larger power output. Increasing the exchange area of the MLCs would also reduce the conduction resistance and thus improve the heat exchange. Finally, it has been shown that MLCs with thicker or more numerous electrodes exchange heat faster, due to the larger thermal conductivity of metals compared to dielectric materials and their connection to the capacitors' outside terminals.^34^ Commercial multilayer capacitors presenting these optimized parameters would thus become key when it comes to improving the performance of heat harvesting devices.

## 4. Conclusion

We presented a study of commercially available multilayer capacitors for non-linear pyroelectric energy harvesting. Among sixteen tested variants, we identified seven potential candidates showing less than 10% losses at 180 °C, with energy densities relative to the total capacitor's volume between 0.3 and 0.8 J cm^−3^ when subjected to an Olsen cycle performed between 25 and 180 °C at 50% of their respective breakdown voltages. The best identified capacitor exhibited an active energy density of 1.3 J cm^−3^ at 250 V. An in-depth characterization revealed that its dielectric material is (Ba,Ca)(Ti,Zr)O_3_, with a Curie temperature of 10 °C. The active volume of the capacitor accounts for 56% of its total volume. By optimizing the pyroelectric cycle around the phase transition and pushing the voltage up to 450 V (*i.e.* 90% of the capacitor's breakdown limit), we showed a maximum active energy density of 3.7 J cm^−3^ for a temperature span of 200 °C. Direct Olsen cycles performed between 0 and 100 °C confirmed the energy density obtained indirectly from polarization measurements under the same conditions, showing 1.1 J cm^−3^ at 400 V. A prospective analysis of how fast heat could be exchanged by these capacitors suggests that a device made of 1000 MLCs of this kind could generate 4 W of electricity. The performance of these commercially available capacitors opens perspectives towards scaling-up, considering that these capacitors are available in very large quantities.

## Author contributions

A. H. wrote the manuscript with E. D. and performed the experimental work. A. E. M. did the SEM analysis that led to Fig. 3a. N. V. performed the SIMS analysis that led to SI Note 4 and helped with the interpretation. C. B. ran the XRD measurement that led to Fig. 3b and helped with the analysis. E. D. and O. R. made valuable contributions to the productive discussions and provided insightful comments. All authors approved the final version of the manuscript.

## Conflicts of interest

The authors declare no conflict of interest.

## Supplementary Material

TA-OLF-D6TA05388B-s001

## Data Availability

The data supporting this article have been included as part of the supplementary information (SI). Supplementary information is available. See DOI: https://doi.org/10.1039/d6ta05388b.
