# Supplementary material for: Non-linear pyroelectric energy harvesting with commercial multilayer capacitors
Source: J Mater Chem A Mater. 2026 Oct 27. Online ahead of print. doi: 10.1039/d6ta05388b (PMC13712173; doi:10.1039/d6ta05388b)
Supplement: Supplementary file 1 [file TA-OLF-D6TA05388B-s001.pdf]

Supplementary Information (SI) for Journal of Materials Chemistry A.
This journal is © The Royal Society of Chemistry

Supplementary material for

NON-LINEAR PYROELECTRIC ENERGY HARVESTING WITH COMMERCIAL MULTILAYER CAPACITORS

Authors:

Amandine Helt [a,b,*], Omar Ramírez [a], Asmaa El Moul [a], Nathalie Valle [a], Cédric Bamberg [a,b], Emmanuel Defay [a,b,*]

Affiliations:

[a] Luxembourg Institute of Science and Technology (LIST), 4362, Esch-sur-Alzette, Luxembourg

[b] University of Luxembourg, 4365, Esch-sur-Alzette, Luxembourg

* Corresponding authors: amandine.helt@list.lu ; emmanuel.defay@list.lu

CONTENT

Supplementary Note 1. QV loops of all screened capacitors 3

Supplementary Note 2. SEM view along (y-z) plane 6

Supplementary Note 3. EDX spectroscopy of electrodes, terminals and dielectric material 7

Supplementary Note 4. SIMS spectrum measured on the dielectric material 8

Supplementary Note 5. Influence of the phase transition on the harvested energy 9

Supplementary Note 6. Comparison of direct and indirect measurements 10

Supplementary Note 7. Biot number calculation 11

Supplementary Table 1. References and measured values for all screened samples 12

Supplementary Note 1. QV loops of all screened capacitors

Hereunder are the QV loops measured with the aixACCT system for all screened capacitors, as explained in section 2.2 of the main article. The analysis of Figure S1-1 and S1-2 led to Figures 2a and 2b, respectively, where harvested energy densities and losses were calculated as explained at the beginning of section 3.1.

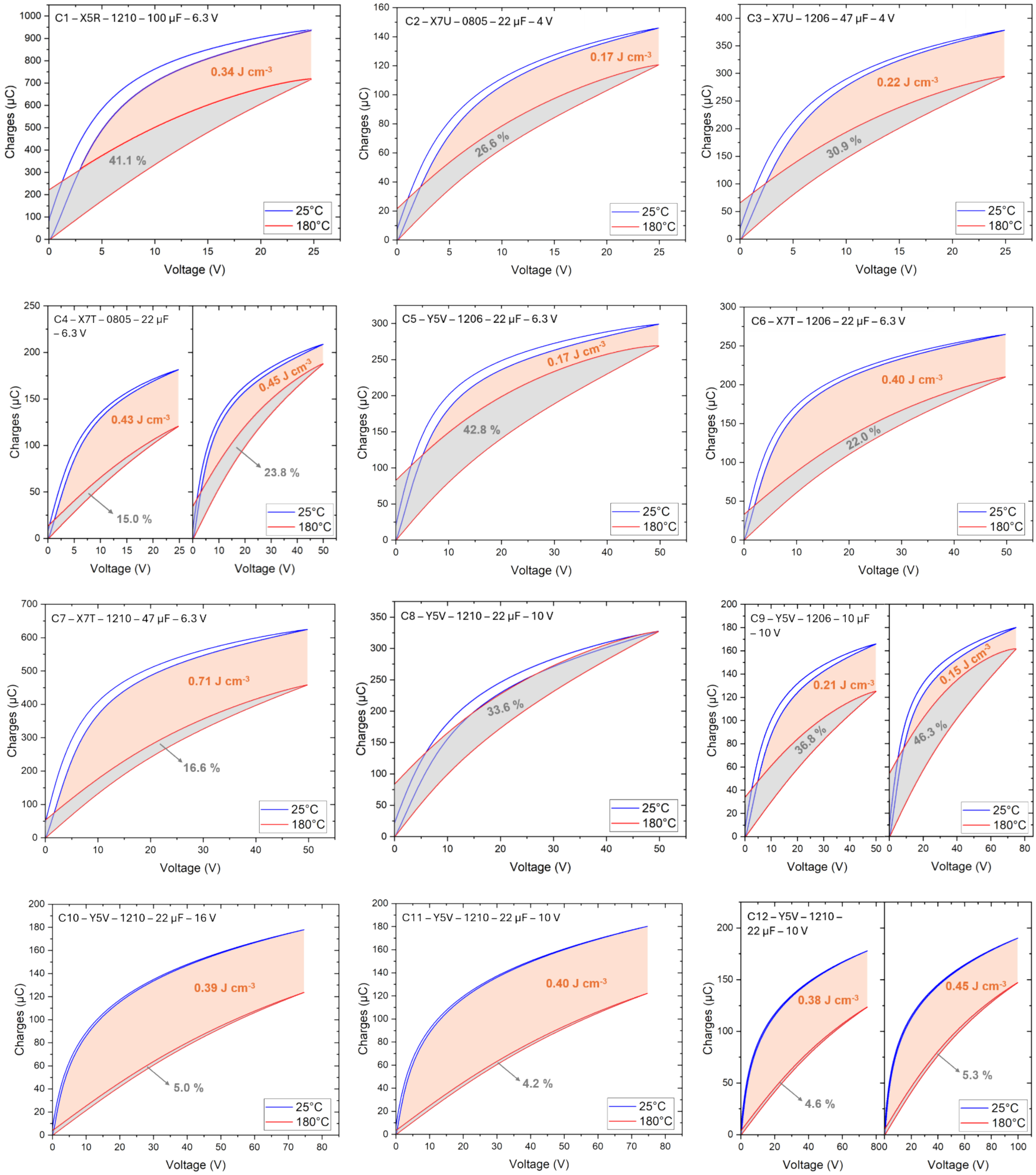

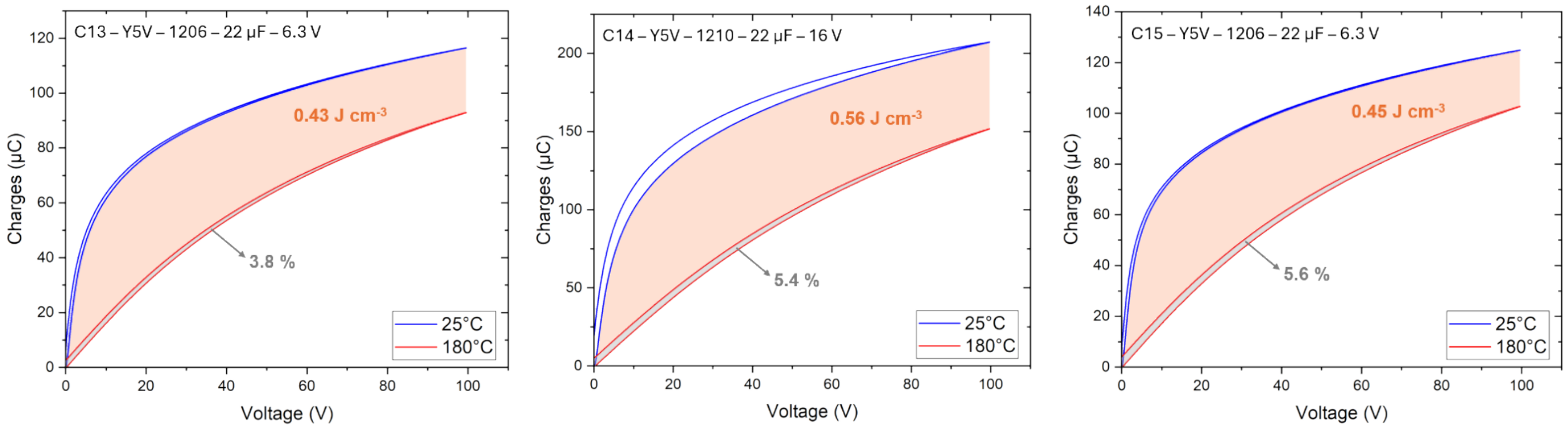

Figure S1-1: Unipolar QV loops measured for all screened capacitors with aixACCT system, for a frequency of 1 Hz, at both 25 °C and 180 °C and under an applied voltage equal to 25% of their respective breakdown voltage. For samples where this voltage falls exactly between two measurements, both are displayed. On each graph, the energy density harvested in one cycle and calculated using the total capacitor's volume is represented by the orange area and given in J cm⁻³. The losses at high temperature are given by the area of the corresponding unipolar loop, here in grey, and are expressed in % of the total energy stored in the capacitor. For samples where two graphs are shown, we consider the average values for the study. A similar chart for sample C16 – Y5V – 1210 – 10 µF – 16 V can be found in the first figure of the main part of the article.

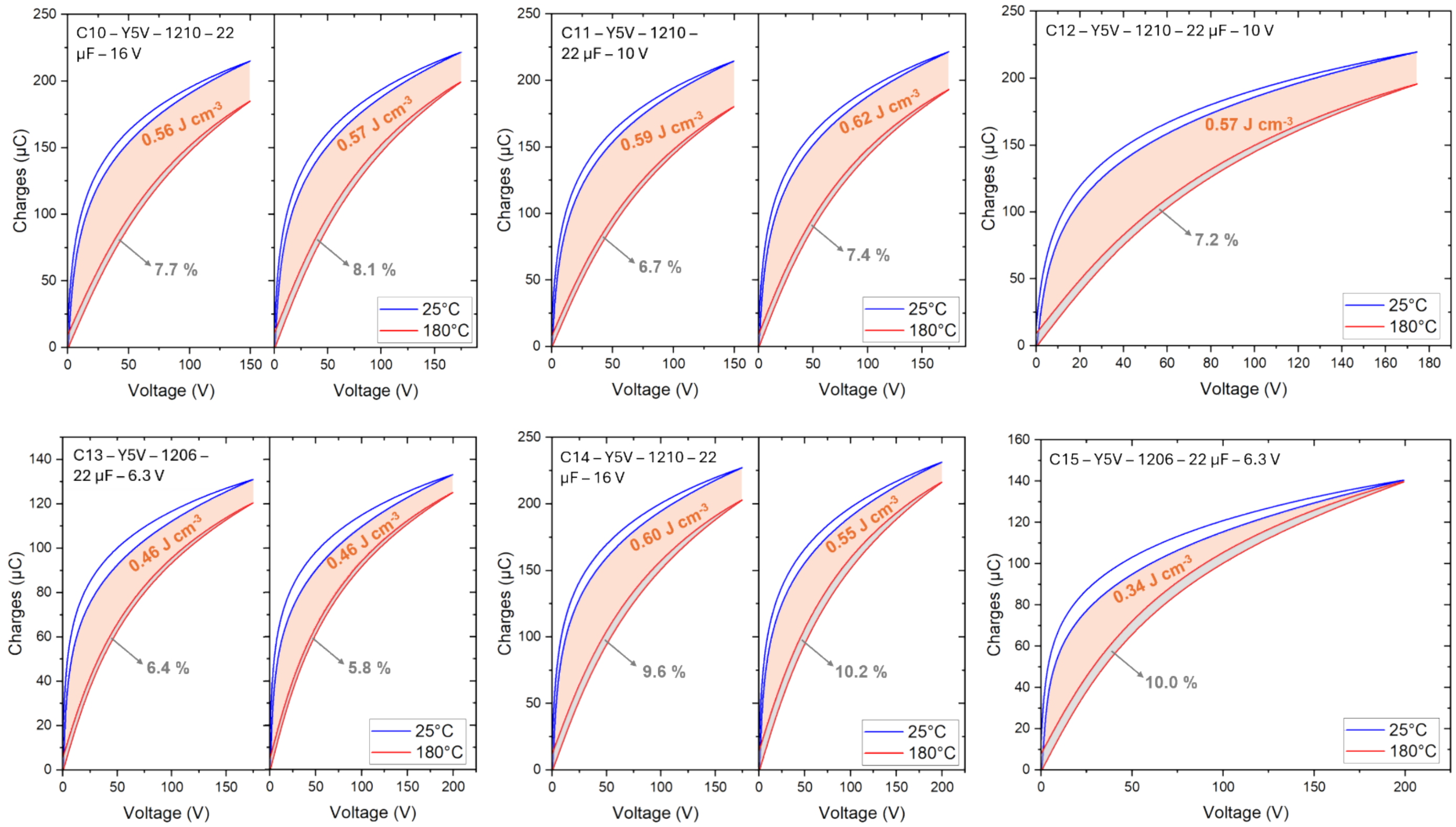

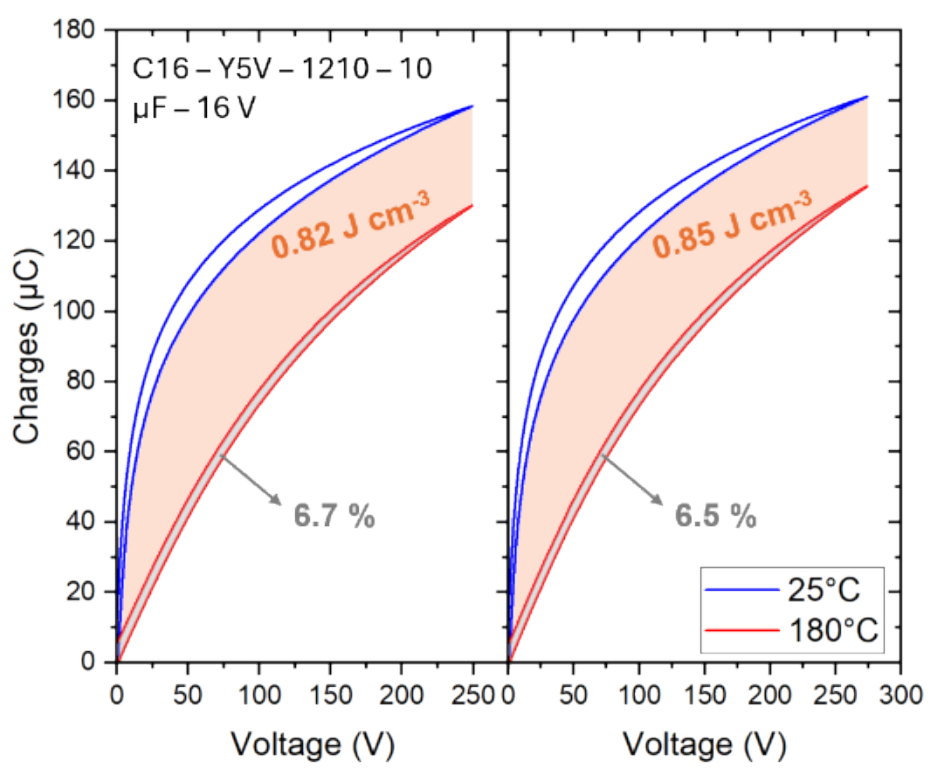

Figure S1-2: Unipolar QV loops measured with aixACCT system on one of the four samples studied for each of the seven best capacitor grades, with a frequency of 1 Hz, at both 25 °C and 180 °C and under an applied voltage equal to 50% of their respective breakdown voltage. On each graph, the energy density harvested in one cycle and calculated using the total capacitor's volume is represented by the orange area and given in J cm⁻³. The losses at high temperature are given by the area of the corresponding unipolar loop, here in grey, and are expressed in % of the total energy stored in the capacitor. For samples where two graphs are shown, meaning when the breakdown voltage falls exactly between two measurements, we consider the average values for the study.

Supplementary Note 2. SEM view along (y-z) plane

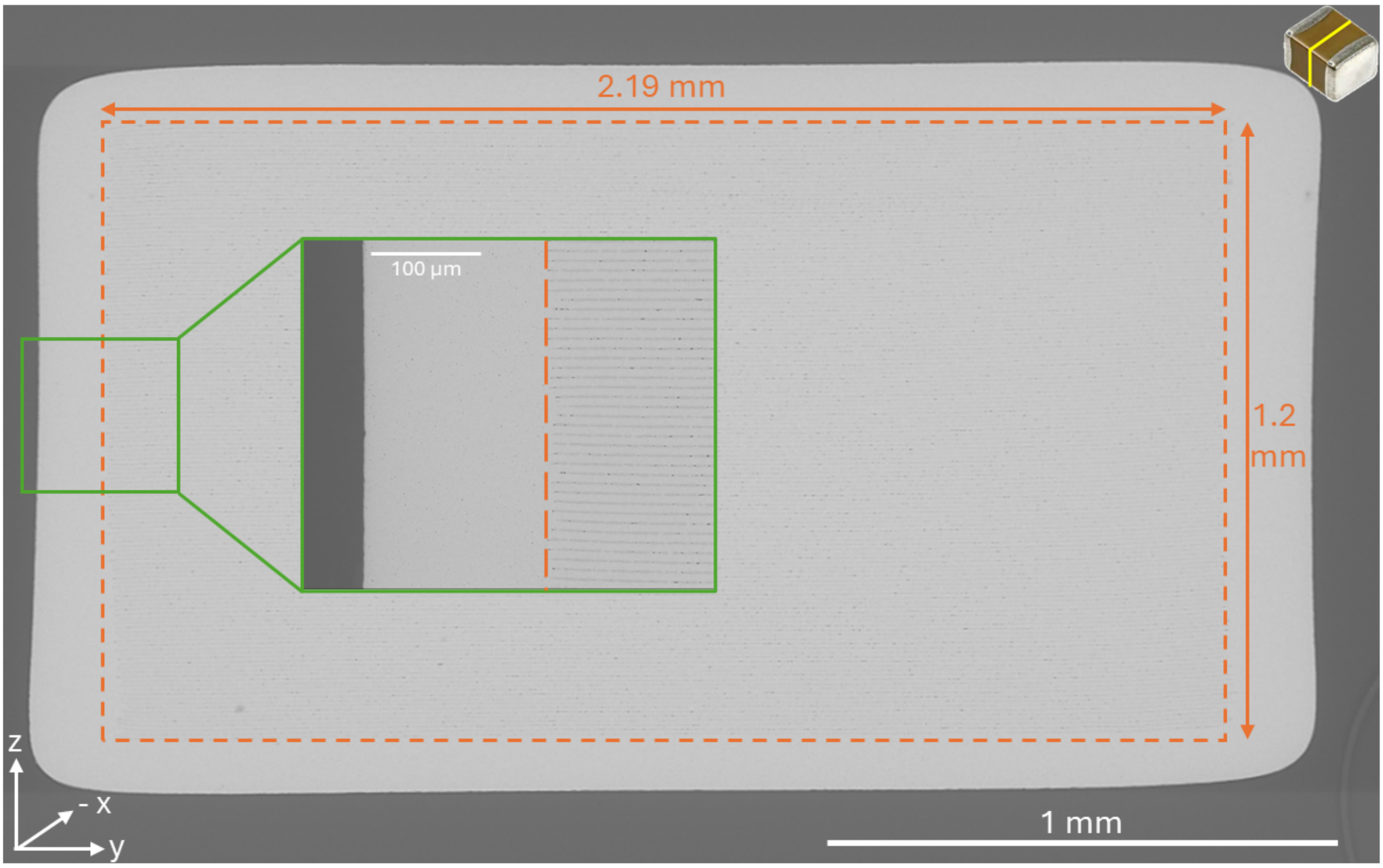

Figure S2: Scanning Electron Microscope (SEM) view of C16 – Y5V – 1210 – 10 µF – 16 V capacitor's cross-section along plane (y-z), as represented in yellow in the top right corner. The zoom-in shows the boundary between the active volume on the right, where electrodes are superimposed, and the dielectric material on the left, which is not involved in energy storage.

Supplementary Note 3. EDX spectroscopy of electrodes, terminals and dielectric material

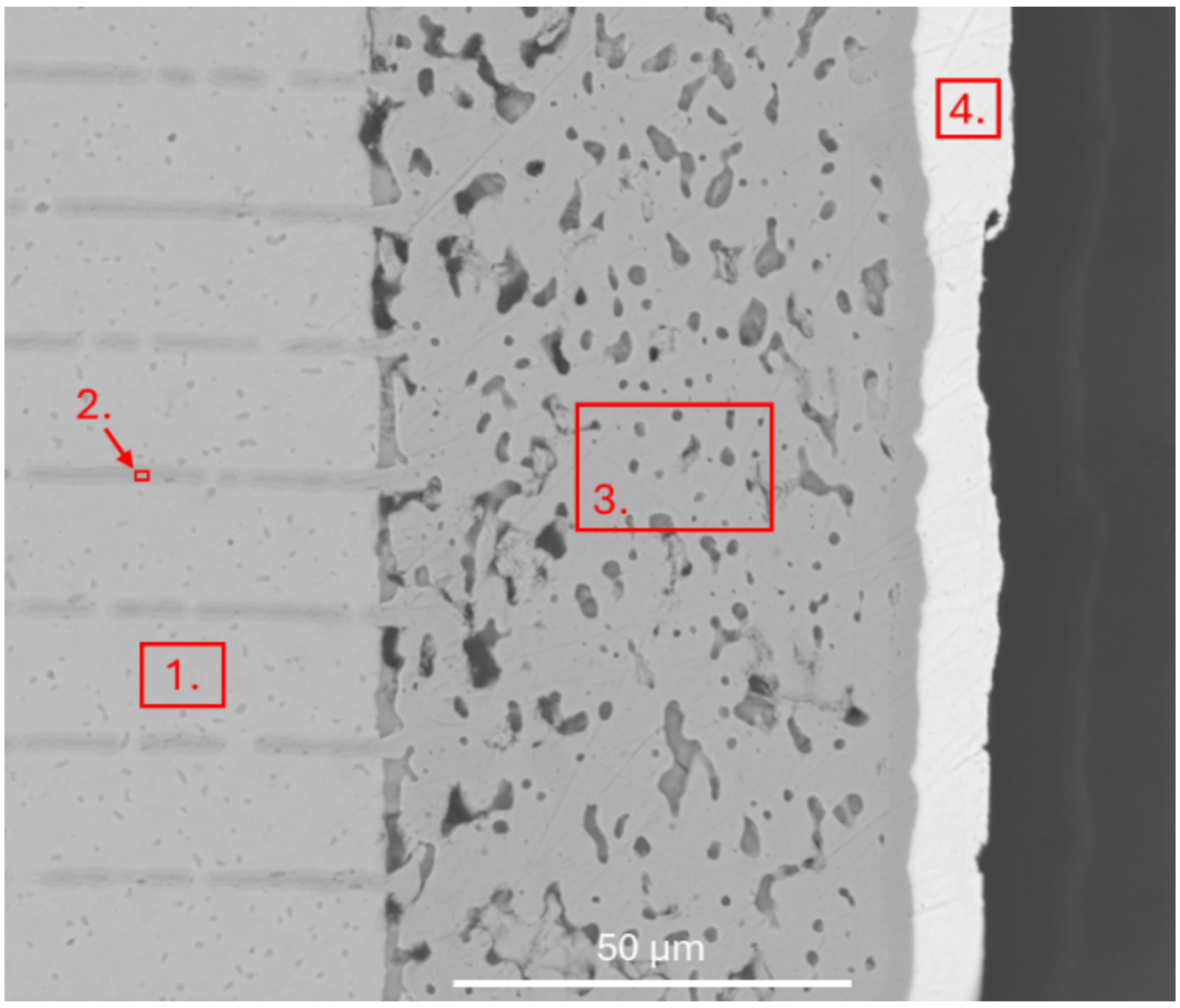

Zone	Ba (at. %)	O (at. %)	C (at. %)	Ni (at. %)	Cu (at. %)	Sn (at. %)	Others (at. %)	Part
1.	**52.48**	**41.20**	/	2.36	/	/	3.96	Dielectric material
2.	3.09	5.32	14.58	**77.01**	/	/	/	Electrode
3.	0.75	5.38	40.5	2.08	**51.29**	/	/	Terminal
4.	/	/	/	**30.66**	3.52	**65.82**	/	Electroplated layer

Figure S3: SEM view of the junction between the dielectric material and the terminal taken on sample C16 – Y5V – 1210 – 10 µF – 16 V. In red are indicated the locations where Energy Dispersive X-Ray (EDX) mappings have been done, whose results are gathered in the table. Numbers in bold indicate the main element(s) of the phase, and the name of the corresponding capacitor's part is given in the last column.

Supplementary Note 4. SIMS spectrum measured on the dielectric material

Hereunder is the mass spectrum measured using SIMS characterization on the dielectric material of sample C16 – Y5V – 1210 – 10 μF – 16 V in order to identify its constituting elements. Intensities for the expected barium and titanium are visible for mass-to-charge ratios around 50 and 135, respectively. Their oxides have also been detected for ratios between 60 and 80, around 155 and above 210, confirming the dielectric material to be a BTO-based ceramic.

Common dopants for BTO are calcium, zirconium and strontium. However, the latter has not been detected, ruling out the hypothesis of a barium strontium titanate (BST) sample. Intensities for Ca and Zr can be seen for ratios of 40 and 90, respectively, meaning that this material is (Ba,Ca)(Ti,Zr)O_3 (BCTZ).

As a caesium primary ion source was used, it is normal to find a peak for this element at around 265. Rubidium (at ratio 85) is also commonly observed when using this type of ion source. The remaining elements, namely sodium (ratio 23), aluminium (at ratio 27) and yttrium (ratio of 89), can be associated with substrate contamination, atmosphere contamination and interferences, respectively.

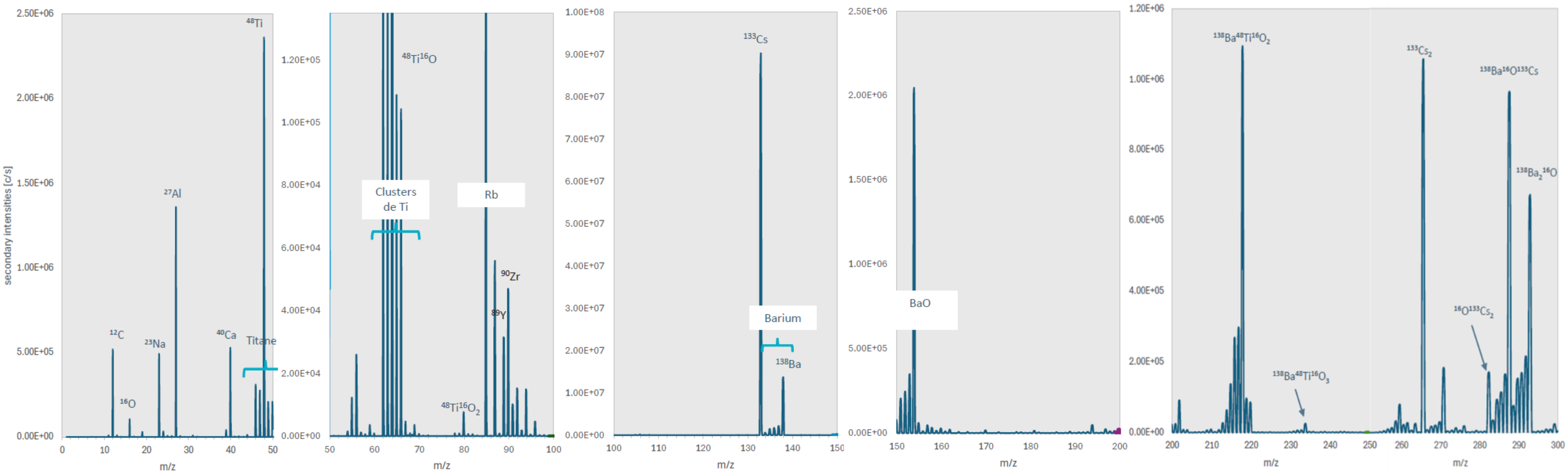

Figure S4: Secondary-Ion Mass Spectrometry (SIMS) spectrum measured on the dielectric material of sample C16 – Y5V – 1210 – 10 µF – 16 V.

Supplementary Note 5. Influence of the phase transition on the harvested energy

Hereunder in Figure S5-1 are the QV loops that were used to draw the temperature-dependent curve of 20 °C energy increments between -50 and 140 °C, represented in Figure 4b.

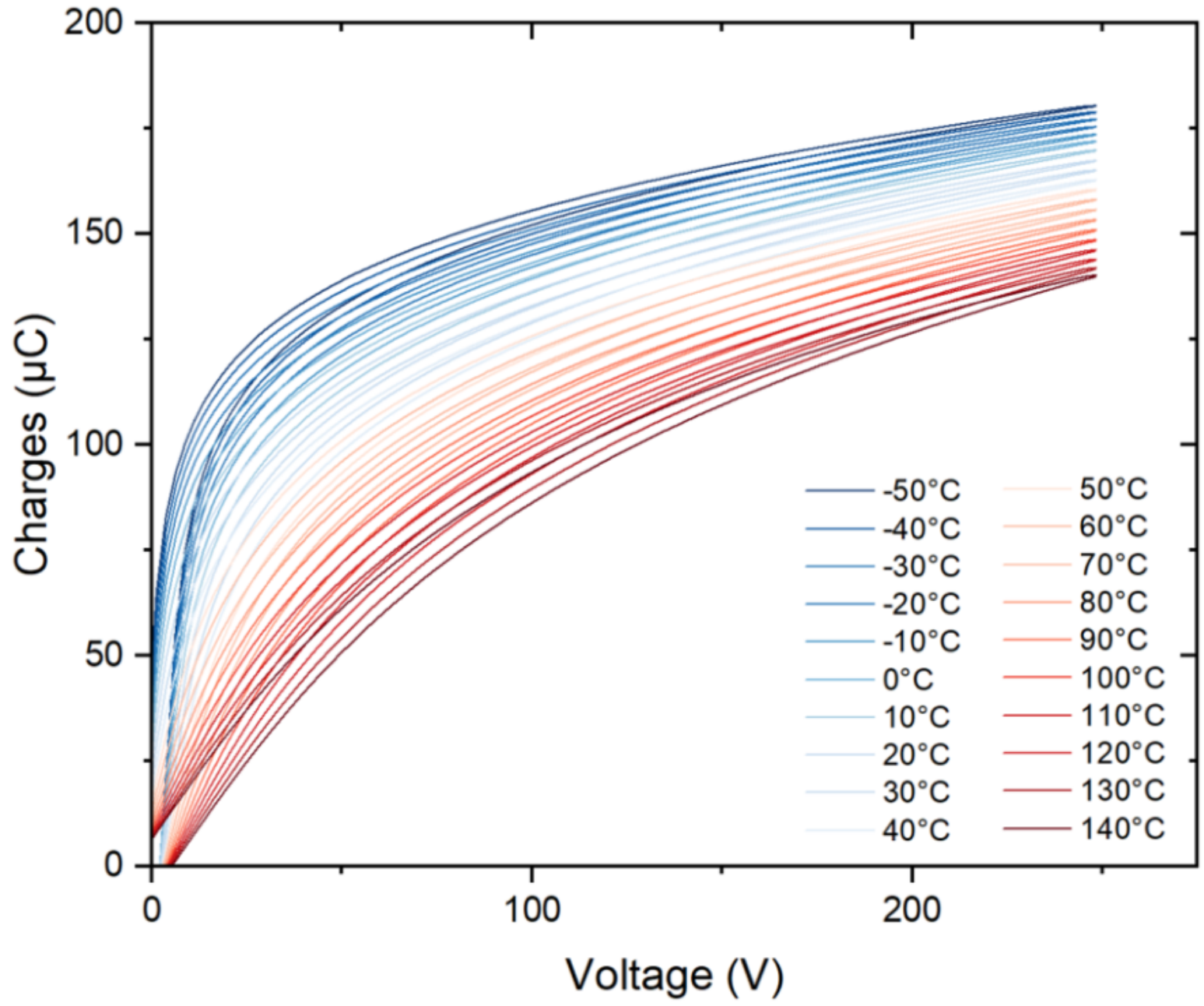

Figure S5-1: Positive part of the bipolar QV loops measured under 250 V, at a frequency of 100 mHz, and for temperatures ranging from -50 to 140 °C.

The following figure shows the QV loops used to extract the maximum harvested energy density and the losses from an Olsen cycle run under 450 V, between -50 and 150 °C. Both values were calculated as explained at the beginning of section 3.1.

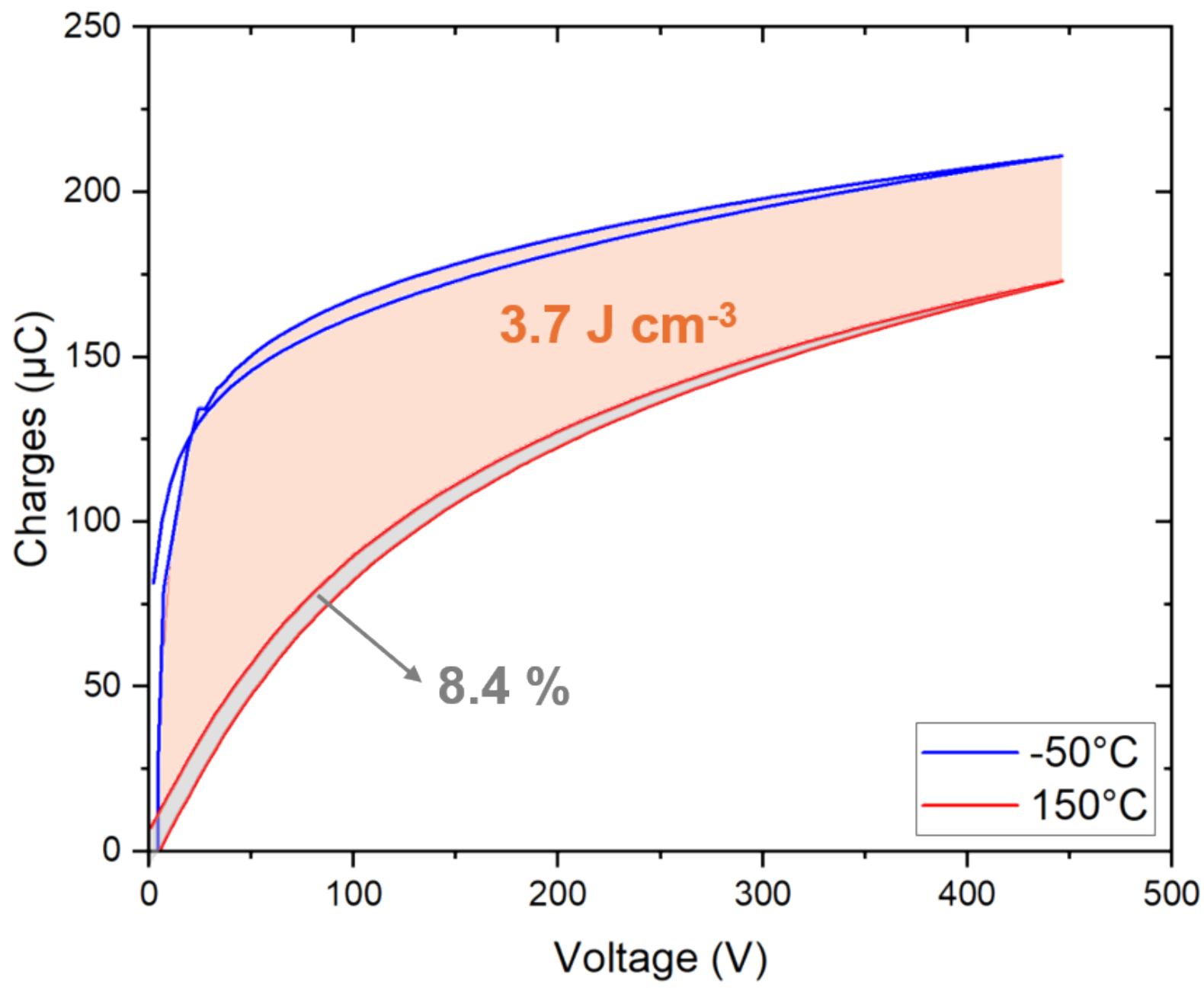

Figure S5-2: Positive part of the bipolar QV loops measured with aixACCT system on capacitor C16 – Y5V – 1210 – 10 µF – 16 V with a frequency of 1 Hz, between -50 °C and 150 °C, under an applied voltage of 450 V. The energy density harvested in one cycle and calculated using the active capacitor's volume is represented by the orange area and given in J cm-3. The losses at high temperature are given by the area of the corresponding unipolar loop, here in grey, and are expressed in % of the total energy stored in the capacitor.

Supplementary Note 6. Comparison of direct and indirect measurements

Hereunder are the graphs representing the indirect (a) and direct (b) measurements performed in order to compare the energy densities harvested through both methods, for an Olsen cycle between 0 and 100 °C at 400 V. The measurement point corresponding to this voltage in the fourth figure of the manuscript stems from Figure S6b.

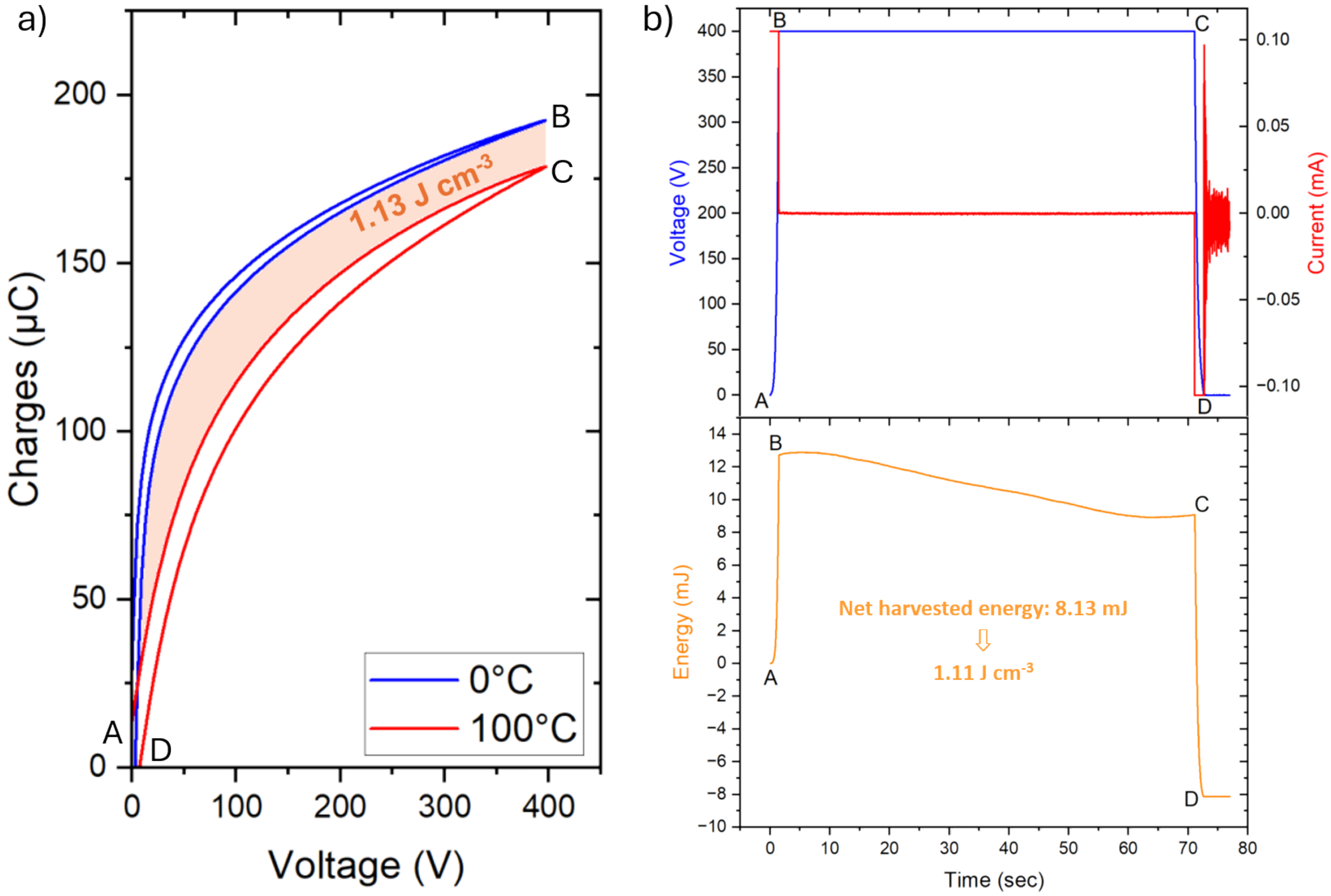

Figure S6: a) Indirect and b) direct Olsen cycles measured on C16 – Y5V – 1210 – 10 μF – 16 V at 400 V between 0 and 100 °C for a frequency of 10 mHz. In b), the upper graph shows the voltage and current applied to the MLC, while the lower graph presents the calculated harvested energy. In both panels, the energy density values were calculated relatively to the active volume of the capacitor and the letters ABCD refer to the different steps of the corresponding Olsen cycle: A-B: MLC charged up to 400 V at 0 °C, B-C: MLC kept at 400 V while the temperature increases up to 100 °C, C-D: MLC discharged at 100 °C, D-A: MLC cooled down to 0 °C at zero voltage.

One can notice that the lower graph of Figure S6b, representing the net energy versus the cycle duration, presents a baseline drift from around 63 s to the end of the cycle, at 72 s. This suggests that the energy that can be harvested during the heating step has reached a limit at 63 s, and that holding the capacitor charged at this high temperature for longer will only lead to leakage. That is why the cycle has been stopped here, to be sure that all the energy possibly harvested from the heating step has been recovered, without losing too much of it due to leakage. The harvested energy in the case of the cycle lasting 63 s (so stopping before the leakage starts) and considering that the C-D step would not change, has been calculated to be 8.29 mJ. This corresponds to an energy density of 1.14 J cm^{-3} calculated using the active volume, which is not much different from the initial 1.11 J cm^{-3} and even closer to the 1.13 J cm^{-3} calculated from the indirect cycles, confirming the accuracy of these measurements.

Supplementary Note 7. Biot number calculation

The Biot number is a dimensionless quantity used to compare the internal conduction resistance of a solid body to the external convection resistance at its surface. It is given by $\mathrm{Bi} = \frac{h\,L_c}{k}$, where k is the thermal conductivity of the material in W m^{-1} K^{-1}, L_c the characteristic length given by the smaller thickness of the body in m, and h the convective heat transfer coefficient in W m^{-2} K^{-1}. For the condition $g << w$, with g the thickness of the channel between two solid bodies and w its length, h is given by $h = \frac{N\,u_D\,k_f}{D_h}$ (Sieder-Tate correlation). The quantity $N\,u_D$ depends on the Graetz number, itself depending on the Reynolds and Prandtl numbers. The equation can be written depending on the known fluid, solid material and channel properties, as: $h = \frac{1.86\left[\frac{2\,Q}{N\,w\,\nu}\,\frac{\nu\,c_{p,f}}{k_f}\,\frac{D_h}{L_{ch}}\right]^{1/3} k_f}{D_h}$, with Q the flow rate, N the number of channels, w their width and L_{ch} their length, ν and μ the fluid kinematic and dynamic viscosities, $c_{p,f}$ the fluid specific heat capacity, k_f the fluid thermal conductivity and $D_h = 2g$ the hydraulic diameter.

In the case of Song *et al.*, the device (GEN3) that led to the generation of 1 W of electrical power is composed of 255 PST MLCs, each 0.5 mm-thick, distributed over 17 columns and 15 rows, thus separated by N = 14 slits of thickness g = 0.42 mm and length L_{ch} = 177 mm. As the width of the MLCs (and thus of the slits) is w = 8 mm, the condition $g << w$ is verified. The power generation experiment has been run using silicon oil at a flow rate Q = 600 mL min^{-1}. The fluid properties, extracted from Song *et al.*'s publication, are given in the following table:

**Property**	**Value**
Kinematic viscosity ν [m^2 s^{-1}]	35 x 10^{-6}
Dynamic viscosity μ [Pa s]	4.5 x 10^{-3}
Specific heat capacity $c_{p,f}$ [J kg^{-1} K^{-1}]	1634
Thermal conductivity k_f [W m^{-1} K^{-1}]	0.16

With these conditions, it is thus possible to calculate a convective heat transfer coefficient of 702 W m^{-2} K^{-1} for this configuration. With the thermal conductivity of PST being equal to 2.1 W m^{-1} K^{-1}, and a characteristic length of 0.5 mm for the MLCs, **the Biot number is thus equal to 0.16**, meaning that the heat transfer is still limited by convection (Bi ≈ 0.1), even if conduction starts having an impact too (Bi >> 1).

Supplementary Table 1. References and measured values for all screened samples

Manufacturer references, measured breakdown voltage and calculated losses and theoretical energy density values for all screened capacitors (associated with Fig. 2).

Sample name	Manufacturer references	Breakdown voltage at 180 °C (V) *	Losses at 180 °C, 25% of BD voltage (%) **	Theoretical energy density between 25 and 180 °C ***, 25% of BD voltage (J cm^{-3}) **	Average losses at 180 °C ± standard deviation, 50% of BD voltage (%) **	Average theoretical energy density ± standard deviation between 25 and 180°C ***, 50% of BD voltage (J cm^{-3}) **
C1 – X5R – 1210 – 100 µF – 6.3 V	12106G107ZAT2A	100	41.1	0.34		
C2 – X7U – 0805 – 22 µF – 4 V	GRM21BE70G226ME51L	100	26.6	0.17		
C3 – X7U – 1206 – 47 µF – 4 V	GRM31CE70G476ME15L	125	30.9	0.22		
C4 – X7T – 0805 – 22 µF – 6.3 V	CM21X7T226M06AT	150	19.4	0.44		
C5 – Y5V – 1206 – 22 µF – 6.3 V	C1206C226Z9VACTU	175	42.8	0.17		
C6 – X7T – 1206 – 22 µF – 6.3 V	GCM31CD70J226KE02L	200	22.0	0.40		
C7 – X7T – 1210 – 47 µF – 6.3 V	GRM32ED70J476KE20L	200	16.6	0.71		
C8 – Y5V – 1210 – 22 µF – 10 V	C1210C226Z8VACTU	225	33.6	N.A.		
C9 – Y5V – 1206 – 10 µF – 10 V	CL31F106ZPHNNNE	250	41.6	0.18		
C10 – Y5V – 1210 – 22 µF – 16 V	1210YG226ZAT2A	342 ± 14	5.0	0.39	7.4 ± 0.7	0.57 ± 0.02
C11 – Y5V – 1210 – 22 µF – 10 V	CC1210ZKY5V6BB226	363 ± 25	4.2	0.40	6.5 ± 0.4	0.58 ± 0.02
C12 – Y5V – 1210 – 22 µF – 10 V	1210ZG226ZAT2A	358 ± 14	4.9	0.42	6.8 ± 0.4	0.58 ± 0.01
C13 – Y5V – 1206 – 22 µF – 6.3 V	CC1206ZKY5V5BB226	381 ± 13	3.8	0.43	5.3 ± 0.6	0.44 ± 0.02
C14 – Y5V – 1210 – 22 µF – 16 V	CC1210ZKY5V7BB226	381 ± 13	5.4	0.56	9.2 ± 0.7	0.54 ± 0.03
C15 – Y5V – 1206 – 22 µF – 6.3 V	12066G226ZAT2A	412 ± 14	5.6	0.45	9.8 ± 0.6	0.35 ± 0.04
C16 – Y5V – 1210 – 10 µF – 16 V	CC1210ZKY5V7BB106	500 ± 20	4.0	0.54	5.3 ± 0.9	0.79 ± 0.04

* For samples C10 to C16, the average over four samples as well as the standard deviation are given

** BD = breakdown *** Calculated relative to the capacitor's total volume
